# Removing a broken guidewire in the hip joint: treatment options and recommendations for preventing an avoidable surgical catastrophe. A case report: Removendo um fio-guia quebrado na articulação do quadril: opções de tratamento e recomendações para prevenir uma evitável catástrofe cirúrgica. Um relato de caso

**DOI:** 10.1590/1516-3180.2014.9061512

**Published:** 2015-10-09

**Authors:** Abhijeet Ashok Salunke, Prem Haridas Menon, Gurunathampalayam Ilango Nambi, Junhao Tan, Vivek Patel, Yongsheng Chen, Jay Kumar

**Affiliations:** I MD. Clinical Fellow, Division of Musculoskeletal Oncology, National University Hospital, Singapore. Assistant Professor, Department of Orthopedics, Pramukswami Medical College, Karamsad, Anand, Gujarat, India; II MD. Clinical Fellow, Department of Orthopedics, National University Hospital, Singapore.; III MD. Consultant, Department of Plastic & Reconstructive Microvascular Services, Kovai Medical Center & Hospital, Coimbatore, Tamil Nadu, India.; IV Medical Student, Yong Lin Loo School of Medicine, National University of Singapore, Singapore.; V MD. Assistant Professor, Department of Orthopedics and Traumatology, Pramukswami Medical College, Karamsad, Anand, Gujarat, India.; VI MD. Resident, Hand & Reconstructive Microsurgery Cluster, Department of Orthopedics, National University Hospital, Singapore.; VII MD. Resident, Department of Orthopedics and Traumatology, Pramukswami Medical College, Karamsad, Anand, Gujarat, India.

**Keywords:** Hip fractures, Pelvis, Hip joint, Bone wires, Bone nails

## Abstract

**CONTEXT:**

Hardware breakage during hip surgery can pose challenging and difficult problems for orthopedic surgeons. Apart from technical difficulties relating to retrieval of the broken hardware, complications such as adjacent joint arthritis and damage to neurovascular structures and major viscera can occur. Complications occurring during the perioperative period must be informed to the patient and proper documentation is essential. The treatment options must be discussed with the patient and relatives and the implant company must be informed about this untoward incident.

**CASE REPORT:**

We report a case of complete removal of the implant and then removal of the broken guidewire using a combination of techniques, including a cannulated drill bit, pituitary forceps and Kerrison rongeur.

**CONCLUSIONS:**

We suggest some treatment options and recommendations for preventing an avoidable surgical catastrophe.

## RESUMO

**CONTEXTO:**

Ruptura de material de síntese durante a cirurgia de quadril pode representar problemas desafiadores e difíceis para cirurgiões ortopédicos. Além de dificuldades técnicas com recuperação do material quebrado, complicações como artrite na articulação adjacente, danos às estruturas neurovasculares e às principais vísceras podem ocorrer. A complicação durante o período perioperatório deve ser informada ao paciente e documentação apropriada é essencial. As opções de tratamento devem ser discutidas com o paciente e seus familiares e a empresa do implante deve ser informada sobre este incidente desagradável.

**RELATO DE CASO:**

Nós relatamos um caso da remoção completa do implante e, em seguida, a remoção do fio-guia quebrado usando uma combinação de técnicas, incluindo uma broca canulada, fórceps da hipófise e *rongeur* de Kerrison.

**CONCLUSÕES:**

Sugerimos algumas opções de tratamento e recomendações para prevenir uma evitável catástrofe cirúrgica.

## INTRODUCTION

Hardware breakage during hip surgery can pose challenging and difficult problems for orthopedic surgeons. Apart from technical difficulties relating to retrieval of the broken hardware, complications such as adjacent joint arthritis and damage to neurovascular structures and major viscera have also been described.^1^ ^4 ^Previously reported techniques for removing broken guidewires in the hip joint include use of over-reaming, use of disc forceps, arthroscopically assisted methods and arthrotomy of the hip joint. In this article, we report a case of complete removal of the implant and then removal of the broken guidewire using a combination of techniques including a cannulated drill bit, pituitary forceps and Kerrison rongeur.

## CASE REPORT

**Figure 1:**
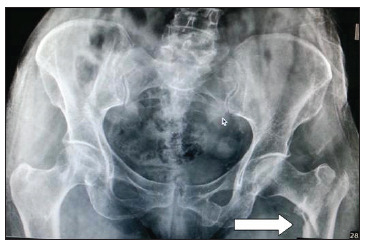
Preoperative radiograph showing Evan's type 4 left intertrochanteric hip fracture (white arrow).

A 65-year-old woman presented with left hip pain following trivial trauma. She was found to have sustained an Evan's type 4 left intertrochanteric hip fracture (Figure 1). The patient had diabetes and hypertension, which were well controlled with oral medication. We decided to fix the fracture surgically using a proximal intramedullary femoral nail. The patient underwent surgery on the third day after the injury.

During the surgery, the patient underwent routine uneventful combined spinal and epidural anesthesia. She was positioned supinely on a standard orthopedic fracture table. Closed reduction of the left intertrochanteric hip fracture was performed under image guidance. The surgeon performed a standard skin incision at 5 cm proximally to the tip of the greater trochanter, in line with the long axis of the femur. The entry point was into the lateral aspect of the greater trochanter, as per our usual practice. After reaming, a short proximal femoral nail was inserted easily. Two guidewires were then placed with the aid of the insertion device, to guide the insertion of the recon screws. A distal hip screw was subsequently placed successfully.

However, during the placement of the proximal hip screw over the cannulated guidewire, a sudden loss of resistance was unexpectedly encountered. Intraoperative images were immediately obtained and the guidewire was found to have broken with its tip lying within the subchondral area of the femoral head. The broken guidewire had not breached the hip joint. The surgeon therefore decided to leave the guidewire alone, with no further attempt at removing it.

**Figure 2:**
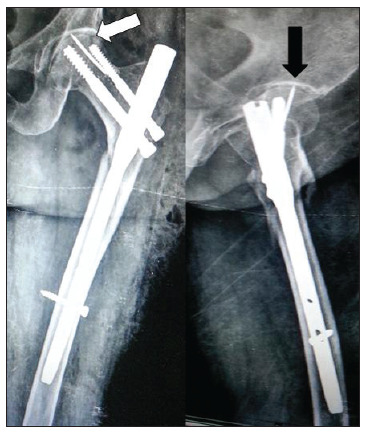
Postoperative radiographs produced after the primary surgery in anteroposterior view (white arrow) and lateral view (black arrow), showing broken guidewire in hip joint and short femoral nail.

**Figure 3:**
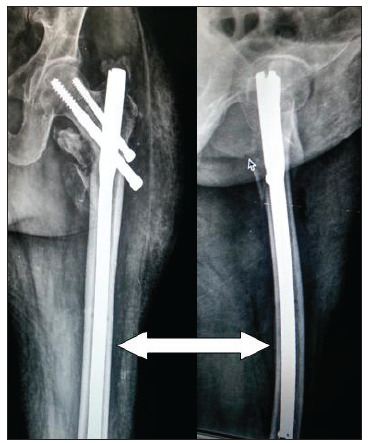
Postoperative radiographs produced after revision

On postoperative day two, plain radiographs were produced, which revealed that the broken guidewire had migrated proximally into the hip joint (Figure 2). The patient therefore underwent a repeat procedure in an attempt to remove the broken guidewire. We began the revision surgery by removing the proximal hip screw. We attempted to reach the broken guide wire with the aid of a cannulated drill bit but we were unable to retrieve the broken wire. We then tried to use the pituitary forceps and Kerrison rongeur to remove the broken guide. Both of the above attempts at removing the broken guidewire were unsuccessful, so we removed the distal hip screw, distal locking screw and intramedullary nail. After complete removal of all other implants, the existing track from the proximal recon screw was enlarged using a cannulated drill bit. We were able to partially engage the tip of the guidewire with the cannulated drill bit. This allowed us to reverse the guidewire slightly into the track. Once the track was large enough, a 2 mm pituitary rongeur and Kerrison rongeur were used successively to grasp the tip of the broken guidewire and remove it. Following successful retrieval of the broken guidewire, a long proximal femoral intramedullary nail was inserted to achieve fracture fixation (Figure 3).

The patient was mobilized with partial weight-bearing on the third postoperative day. Her postoperative recovery was uneventful and she was discharged five days after the revision procedure. She underwent routine follow-up in the outpatient clinic. By three months after the operation, her fracture had completely united and she was walking independently without support.

## DISCUSSION

We searched for similar cases in different databases (PubMed and Lilacs database) using the terms: "hip joint" AND "guide wire" AND "breakage" (Table 1). We found that few cases have been published. Abstracts or full texts were analyzed and it was seen that less than 20 reports were similar to ours (Table 2).^4^ ^9 ^This literature review suggests that the most commonly broken orthopedic hardware is the drill bit, followed by guidewires. The complications caused by broken hardware include adjacent joint arthritis and damage to neurovascular surgery, showing that the broken guidewire was removed and a long femoral nail was inserted (white arrow). structures and major viscera. Hardware may break through reuse, or due to technical problems such as drilling at a low angle of incidence or inadvertent bending during insertion. Price et al. and Sharma et al. studied the reasons for hardware failure due to repeated use of guidewires. They suggested that repeated use of guidewires over time causes deformation and loss of bending and torsional strength, such that the chances of failure increase while drilling and inserting a screw over the wire.^5^ ^6 ^In addition, technical errors that predispose towards intraoperative guidewire breakage may be due to inadvertent changes in direction and twisting during insertion, penetration of the subchondral bone into the hip joint, or insertion of a reamer or screw improperly at an angle to the guidewire, thereby causing excessive contact stress.

The known methods of removing of broken guidewires include use of over-reaming, use of pituitary forceps, arthroscopically assisted methods of retrieval and open arthrotomy and dislocation of the hip joint.^6^ ^9^

It has been shown that the cross-sectional shape of the guidewire changes from round to oval following breakage.^9^ This was perhaps the reason why we were able to use the cannulated drill bit to engage the broken guide wire, to assist in its removal in the present case.

**Table 1:** Results from review of the literature in medical databases for case reports and case series regarding hip fractures with implant breakage. Search conducted on September 10, 2014

Database	Search strategies	Papers found	Papers related
Medline (Via PubMed)	((bone wires) OR (guide wire) OR (guide wires)) AND (hip joint)	27	9
Lilacs database	((bone wires) OR (guide wire) OR (guide wires)) AND (hip joint)	35	7

**Table 2:** Review of the literature regarding implant removal from hip joint

Author	Year	Method of removing broken implant from hip joint	Comments
Sharma et al.^6^	2008	Dynamic hip screw reamer	
Sen et al.^7^	2010	Laparotomy	Two cases of implant breakage, in hip joint and pelvis respectively
Peivandi et al.^9^	2011	Cannulated drill bit	
Current study	2014	Implant removal using pituitary rongeur, Kerrison rongeur and cannulated drill bit	A combination of methods was used for removing the broken implant

Another technique that has been described is that of overreaming. This method uses a larger reamer, such as that of a dynamic hip screw reamer, to provide access to the broken guidewire. The main disadvantage of over-reaming is that it leads to formation of large bone defects, which predispose towards development of pathological fractures.

The method that we used in the present case included firstly complete removal of the implant and then removal of the broken guidewire using a combination of techniques including a cannulated drill bit, pituitary forceps and Kerrison rongeur. The disadvantage of our technique was that removal of the implant led to loss of the reduction of the fracture. Fortunately, the fracture pattern was readily amenable to closed reduction and therefore did not pose much difficulty. A long femoral nail was used for re-fixation, in order to achieve better stability.

Finally, arthrotomy and hip dislocation is the last resort for removal of broken guidewires. The complications from this method include loss of fracture reduction, avascular necrosis of the hip and an increased chance of recurrent hip dislocation, especially if an extensive approach is used.

Complications that occur during the perioperative period must be informed to the patient and proper documentation is essential. The treatment options must be discussed with the patient and relatives.

Furthermore, the implant company must be informed about this untoward incident.^10^

### Clinical recommendations

- Use new guidewires for every new case of nailing of long bones.
- Avoid unintended changes of direction and bending during insertion of the guidewire, reaming and screw insertion.
- Give proper counseling to the patient and relatives about perioperative complications and their consequences and treatment.
- Produce proper documentation of perioperative complications and report the occurrence to the implant company.

## CONCLUSIONS

The methods for retrieval of broken guidewires in the hip joint include use of over-reaming, use of pituitary forceps, use of Kerrison rongeur, arthroscopically assisted methods, complete removal of implant prior to retrieval of broken hardware and arthrotomy of the hip joint. Occasionally, a combination of the above techniques has to be used to achieve successful removal of the broken hardware.
